# Honokiol in cancer: Roles in enhancing combination therapy efficacy and preventing post-transplant malignancies

**DOI:** 10.36922/td.8152

**Published:** 2025-05-05

**Authors:** Laxminarayan Rawat, Raghu Solanki, Rahul Kumar, Soumitro Pal, Akash Sabarwal

**Affiliations:** 1 Division of Nephrology, Boston Children’s Hospital, Boston, Massachusetts, United States of America; 2 Harvard Medical School, Boston, Massachusetts, United States of America; 3 Department of Biological Sciences and Engineering, Indian Institute of Technology Gandhinagar, Palaj, Gujarat, India; 4 Dr. B. R. Ambedkar Institute Rotary Cancer Hospital, All India Institute of Medical Sciences, New Delhi, India

**Keywords:** Honokiol, Cancer, Combination therapy, Chemotherapy, Receptor tyrosine kinase inhibitors, Post-transplantation cancer

## Abstract

Therapeutic resistance remains a significant challenge in cancer treatment, often resulting in relapse and poor outcomes. Conventional chemotherapies, such as cisplatin and paclitaxel, are frequently undermined by the development of chemoresistance and systemic toxicity. Targeted therapies, such as receptor tyrosine kinase (RTKs) inhibitors and monoclonal antibodies (mAbs), offer better specificity but face resistance over time. Combination therapies are being explored to improve efficacy and mitigate resistance. Honokiol, a biphenolic natural compound derived from *Magnolia* species, has emerged as a potential adjunct in combination therapies due to its anti-cancer, anti-inflammatory, and immunomodulatory properties. It enhances the efficacy of chemotherapies, such as cisplatin and paclitaxel, RTK inhibitors, such as cabozantinib and erlotinib, and mAbs, such as cetuximab. Notably, honokiol combined with mAbs has shown promise in pre-clinical studies by reactivating the immune system and reducing tumor growth in resistant models. In addition, honokiol aids in post-transplant cancer prevention by modulating immune responses, reducing tumor progression, and lowering the required dose of immunosuppressants, such as cyclosporine A and rapamycin. Pre-clinical studies in renal cell carcinoma (RCC), head and neck squamous cell carcinoma (HNSCC), and non-small cell lung cancer emphasize its potential to overcome resistance. Despite promising evidence, further clinical studies are needed to validate honokiol as a viable adjunct in combination therapies. While several reviews have focused on the effects of honokiol alone, there is a lack of comprehensive studies examining its potential in combination with other therapies. This review aims to fill this gap by offering critical insights into the role of honokiol as a candidate for combination therapy.

## 1. Introduction

Therapeutic resistance is a significant barrier to achieving durable responses in cancer treatment.^1^ Despite considerable advances in the development of chemotherapies, targeted therapies, and monoclonal antibodies (mAbs), resistance, both intrinsic and acquired, continues to drive treatment failure, tumor progression, and poor patient prognosis.^2^ Conventional chemotherapeutic agents, such as paclitaxel and doxorubicin, have long been the cornerstone of cancer management. However, their non-specific cytotoxicity often results in dose-limiting toxicities and the emergence of resistant tumor clones.^3,4^

The introduction of molecular targeted therapies, particularly receptor tyrosine kinase (RTKs) inhibitors, marked a pivotal advancement in precision oncology.^5^ Drugs, such as cabozantinib, lapatinib, erlotinib, and osimertinib, selectively inhibit key oncogenic drivers in various malignancies. However, resistance to these agents often develops through secondary mutations, bypass signaling, and activation of compensatory pathways. Similarly, mAbs such as cetuximab have transformed the treatment landscape of many cancers. Nevertheless, immune escape mechanisms and tumor microenvironment factors frequently limit their long-term efficacy.

**Figure 1.**
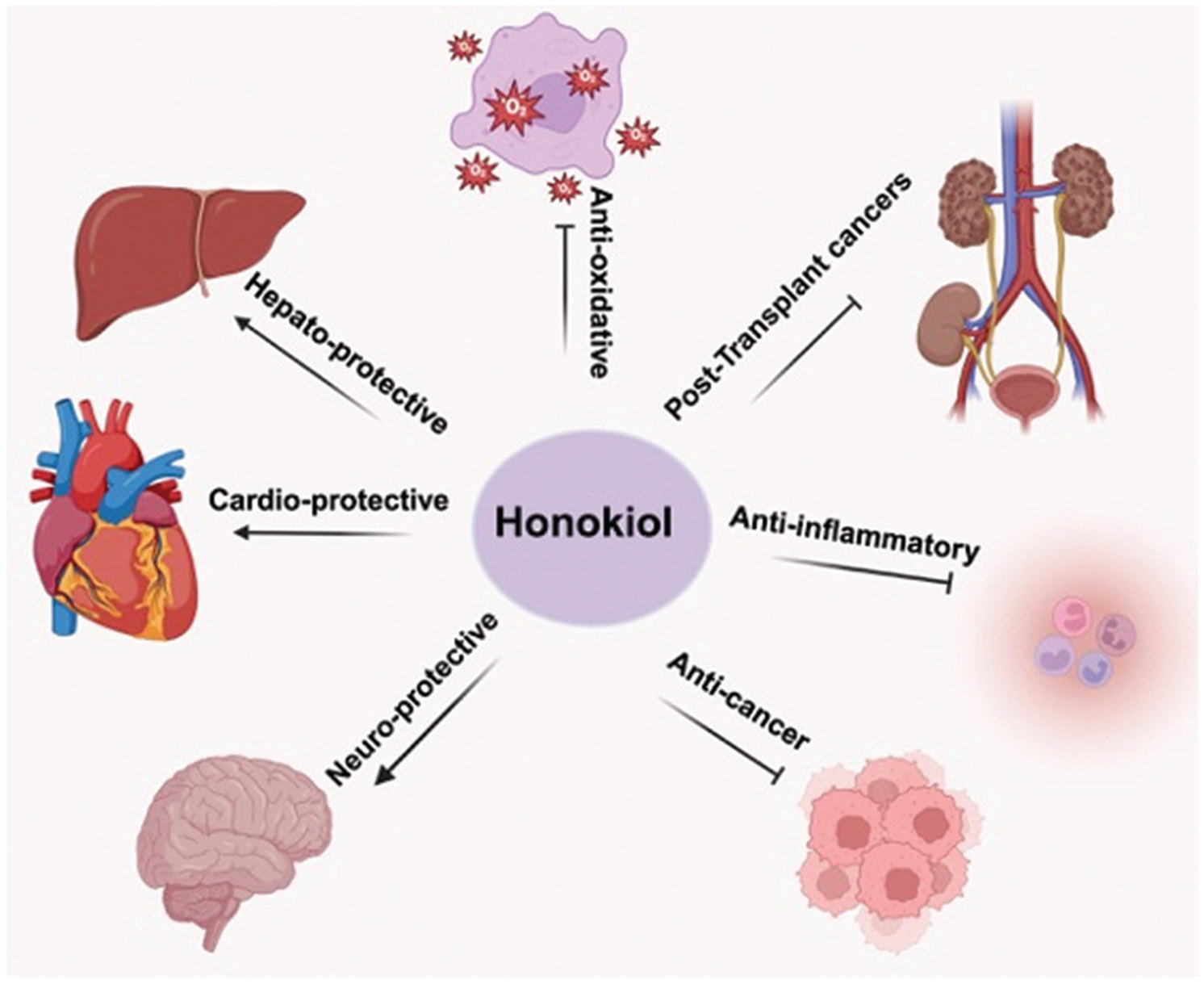
The figure illustrates the diverse biological activities of honokiol with broad therapeutic potential. Adapted and modified from our previously published article (*Phytochemistry Reviews,* 2025, Solanki *et al.*^18^), with copyright permission and license obtained from Springer Nature (Licence Number: 5986590534892).

Combination therapies are increasingly recognized as a strategic approach to overcoming therapeutic resistance.^6,7^ In this context, bioactive natural compounds have gained significant interest due to their multitargeted actions, favorable safety profiles, and the ability to synergize with standard therapies.^8-14^ Honokiol, a biphenolic compound derived from the bark and leaves of the *Magnolia* species, has demonstrated a broad spectrum of pharmacological properties, including anti-cancer, anti-inflammatory, antioxidant, and neuroprotective effects (Figure 1).^15-18^ Importantly, honokiol has shown the potential to resensitize resistant cancer cells to chemotherapeutic agents and targeted therapies, while enhancing the efficacy of mAbs.^19^ Mechanistically, honokiol modulates several key oncogenic and survival pathways, including phosphoinositide 3-kinase (PI3K)/protein kinase B (AKT), mitogen-activated protein kinase (MAPK)/extracellular signal-regulated kinase, signal transducer and activator of transcription 3 (STAT3), and nuclear factor kappa B (NF-κB), and can reverse epithelial-mesenchymal transition, inhibit angiogenesis, and restore immune surveillance.^20^

In addition to its role in restricting cancer cell proliferation, honokiol has emerged as a promising candidate for preventing post-transplantation malignancies. Immunosuppressive agents, such as cyclosporin A and rapamycin, commonly used to prevent graft rejection, can paradoxically promote tumorigenesis by suppressing immune surveillance and activating oncogenic pathways.^21,22^ Honokiol, when combined with these immunosuppressants, has demonstrated efficacy in mitigating cancer-promoting signals while maintaining graft viability in pre-clinical models. This review aims to summarize the present pre-clinical evidence on honokiol, focusing on its role in combination therapies for cancer treatment, where its dual anti-inflammatory and anti-tumor effects may offer significant benefits.

## 2. Combination therapies with honokiol

**Figure 2.**
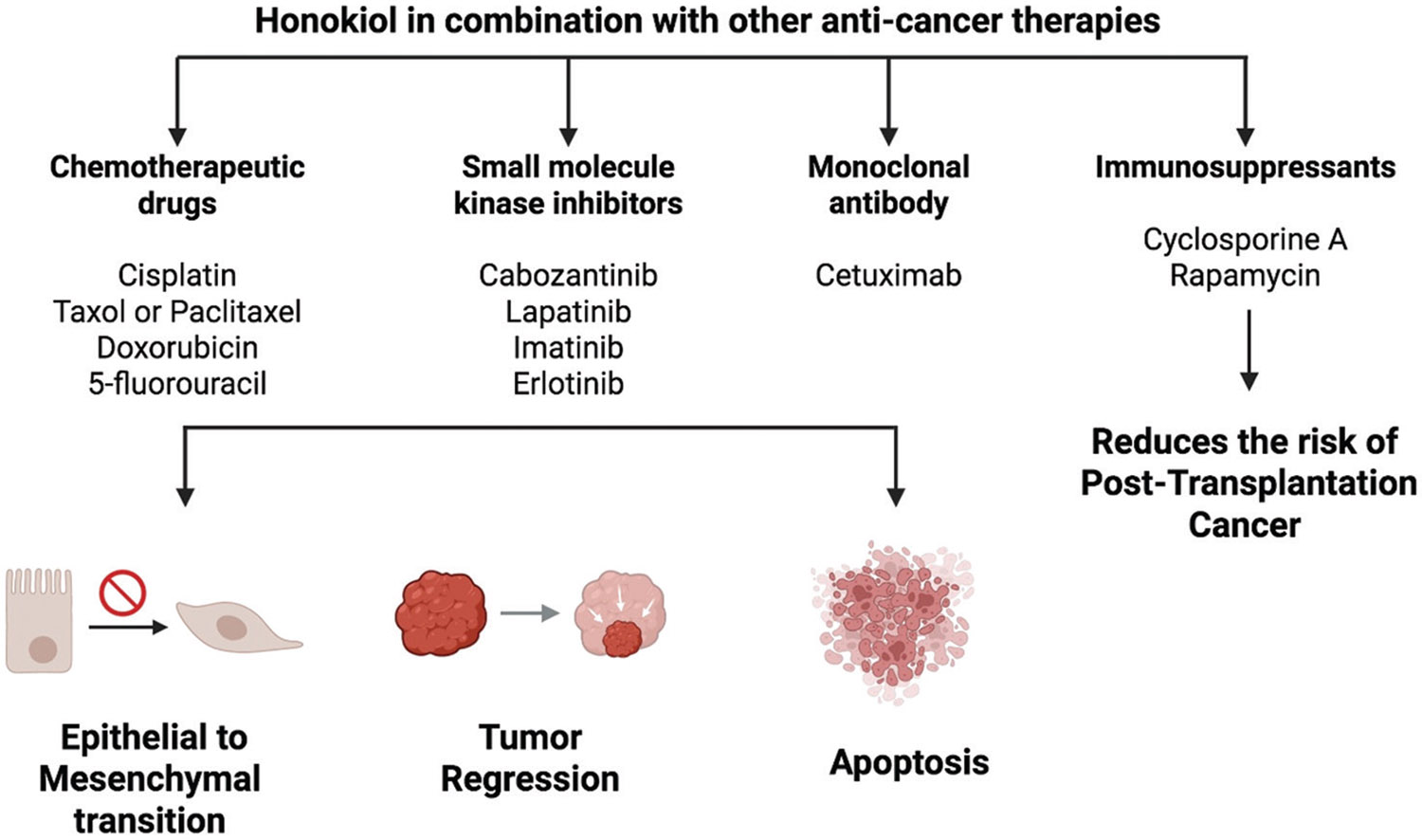
Summary of pre-clinical therapeutic combinations with honokiol. Image created in BioRender. Sabarwal, a. (2025) https://BioRender.com/i48w904.

Combination therapy involving honokiol has been extensively investigated in pre-clinical studies (Figure 2). Both *in vitro* and *in vivo* research have demonstrated that honokiol can enhance the efficacy of chemotherapy, radiation therapy, and targeted therapies across various cancers, including renal, oral, breast, lung, pancreatic, and colorectal cancer.^23-26^ These studies suggest that honokiol could improve treatment outcomes when combined with conventional therapies.

### 2.1. Cisplatin and honokiol

Cisplatin, a widely used chemotherapy drug, is effective against cancers, such as ovarian, bladder, lung, and testicular cancer.^27^ It damages DNA in cancer cells, preventing their division and proliferation. However, cisplatin’s clinical use is limited by side effects such as nausea, kidney damage, hearing loss, and nerve toxicity. To overcome these limitations, cisplatin is often used in combination with other agents.

Pharmacological studies indicate that combining cisplatin with honokiol significantly enhances its therapeutic efficacy while reducing side effects, particularly in colorectal and ovarian cancer.^28,29^ For instance, honokiol has been shown to regulate interleukin 6 (IL-6)/STAT3 signaling pathway in oral carcinoma stem cells, sensitizing cancer cells to cisplatin.^30^ In addition, when combined with curcumin, honokiol sensitizes multidrug-resistant lung cancer cells to cisplatin.^31^

A major limitation of cisplatin is nephrotoxicity. However, studies have demonstrated that honokiol pretreatment reduced cisplatin-induced cytotoxicity by improving cell viability and reducing lactate dehydrogenase release. Honokiol also mitigates oxidative stress by reducing reactive oxygen species (ROS) and enhancing antioxidant enzyme activity in renal epithelial cells.^32^ Honokiol has also been shown to protect against cisplatin-induced acute kidney injury in animal models by modulating mitochondrial fission and regulating key proteins such as dynamin-related protein 1 (DRP1)^33^ and sirtuin 3.^34^ Moreover, honokiol enhances therapeutic responses when combined with carboplatin and gemcitabine in docetaxelresistant tumors.^19^

### 2.2. Paclitaxel (Taxol) and honokiol

Paclitaxel, a taxane chemotherapy agent, stabilizes microtubules and prevents cell division.^35^ It is widely used to treat various cancers, including breast, ovarian, and lung cancers. The combination of paclitaxel and honokiol has been studied with promising results in several cancer models. A study by Wang *et al.*^36^ demonstrated that the combination of honokiol and paclitaxel synergistically affected the multidrug-resistant human squamous KB cells *in vitro*. This combination also significantly inhibited tumor growth in a subcutaneous model, which was accompanied by a decrease in antigen Kiel 67 expression (a marker of cell proliferation) and an increase in terminal deoxynucleotidyl transferase dUTP nick end labeling-positive cells (indicative of apoptosis).^32^ In lung cancer models, the combination of honokiol and paclitaxel-induced significant cell death in both sensitive (H1650, H1299) and resistant (H1650/PTX) cells through paraptosis, a form of programmed cell death involving vacuolation. This effect was observed in both *in vitro* and xenograft tumor models.^37^

Further studies have shown that dequalinium-modified paclitaxel combined with honokiol micelles exhibits promising therapeutic effects against non-small-cell lung cancer (NSCLC).^38^

The combination suppressed vasculogenic mimicry channels and tumor metastasis by activating apoptotic enzymes such as caspase-3 and caspase-9, and down-regulating key pathways, such as focal adhesion kinase, PI3K, matrix metallopeptidase (MMP)-2, and MMP-9. *In vivo* data revealed selective accumulation of these micelles at tumor sites, providing targeted antiproliferative effects.^38^

Wang *et al.*^38^ also explored the use of pH-sensitive polymeric micelles to co-encapsulate paclitaxel and honokiol, achieving suppression of multidrug resistance and metastasis in breast cancer cells. The micelles reversed multidrug resistance by down-regulating P-glycoprotein expression and increasing plasma membrane fluidity.^39^ Similarly, Lu *et al.*^39^ demonstrated that paclitaxel combined with honokiol nanosuspensions, encapsulated in thermosensitive hydrogels, allowed for sustained and targeted drug release at the tumor site.^40^ Honokiol has also shown benefits as a complementary therapy in patients with paclitaxel-resistant tumors, particularly when administered intravenously.^19^

### 2.3. Doxorubicin and honokiol

Doxorubicin, a potent chemotherapeutic agent used for advanced-stage cancers, is known for its high toxicity, particularly cardiotoxicity.^41^ To mitigate these effects, doxorubicin is often administered in combination with other agents to enhance efficacy while reducing toxicity.^42,43^ Studies have investigated the combination of doxorubicin and honokiol, which has shown promise in complementing doxorubicin’s anticancer effects while mitigating cardiotoxicity. Honokiol has been shown to reverse doxorubicin resistance in human breast cancer cells by targeting a molecular pathway involving microRNA-188-5p, *FBXW7*, and *c-Myc*. Honokiol increases the expression of microRNA-188-5p, which upregulates *FBXW7*, a tumor suppressor gene that downregulates *c-Myc*, effectively reversing drug resistance and inhibiting tumor growth.^44^ Moreover, honokiol enhances doxorubicin’s efficacy by regulating mucin 1 and multidrug resistance protein 1, further improving its therapeutic effects and reducing the likelihood of resistance.^45^ Importantly, honokiol’s cardioprotective properties provide a significant advantage, offering a safer combination therapy for patients receiving doxorubicin.

### 2.4. 5-Fluorouracil (5-FU) and honokiol

5-FU, a pyrimidine analog, is a widely used chemotherapeutic agent that inhibits nucleic acid synthesis, thereby suppressing cancer cell growth and proliferation. However, its clinical utility is often limited by toxicity and resistance.^46^

Several studies have explored the combination of 5-FU with honokiol, demonstrating enhanced efficacy and reduced side effects. Ji *et al.*^46^ investigated this combination in oral squamous cell carcinoma cells and *in vivo* models. Their findings revealed that the combination induced significantly higher levels of apoptosis and suppressed tumor growth more effectively than either agent alone.^47^

Similarly, honokiol induced apoptosis in human urothelial cell carcinoma cells and caused G0/G1 cell cycle arrest. When combined with 5-FU, honokiol exhibited a synergistic effect, further enhancing the therapeutic response.^48^ Swidan *et al.*^48^ reported that combining 5-FU and nanoparticulated honokiol significantly reduced tongue carcinoma induced by 4-nitroquinoline oxide in Wistar albino rats. Notably, this combination therapy also decreased systemic toxicity compared to either treatment alone.^49^ These findings suggest that honokiol can potentiate the anti-tumor effects of 5-FU while mitigating its adverse effects, making it a potential adjunct in cancer therapy.

### 2.5. Metformin and honokiol

Metformin, an established anti-diabetic medication, has gained attention for its potential anticancer effects. By lowering systemic glucose levels, metformin limits the energy supply available to cancer cells, thereby inhibiting their growth and proliferation. Studies have shown that combining metformin with honokiol yields promising synergistic effects. In hormone-resistant breast cancer cells (MCF7/HT), the combination of honokiol and metformin effectively inhibited cell proliferation and induced apoptosis. This suggests that the dual treatment may overcome resistance mechanisms common in hormoneindependent breast cancers, enhancing therapeutic efficacy.^50^ These findings highlight the potential of honokiol and metformin as a combination strategy to exploit metabolic vulnerabilities in cancer cells. Further research could establish this regimen as a viable therapeutic approach for hormone-resistant cancers.

### 2.6. Bleomycin and honokiol

Bleomycin is an important chemotherapeutic agent used in the treatment of Hodgkin lymphoma and testicular germ-cell tumors, two of the most curable cancers. However, its clinical application is frequently limited by serious pulmonary side effects, including hypersensitivity pneumonitis, bronchiolitis obliterans organizing pneumonia, acute interstitial pneumonia, and progressive pulmonary fibrosis.^51^ Combining honokiol and bleomycin has enhanced anticancer efficacy while potentially reducing toxicity. In breast cancer (MCF7), pancreatic cancer (PANC-1), and melanoma (UACC903) cell lines, honokiol reduced the effective concentration of bleomycin by tenfold. This enhanced potency is attributed to honokiol’s ability to inhibit the repair of bleomycin-induced single- and doublestrand DNA damage, thereby promoting cancer cell death. By enabling lower therapeutic doses of bleomycin, this combination may help minimize pulmonary side effects while maintaining or improving anticancer activity.^52^ These findings suggest that honokiol could serve as an effective adjuvant to bleomycin-based chemotherapy.

## 3. Monoclonal antibody and honokiol combination

Monoclonal antibodies have revolutionized cancer therapy by targeting tumor-associated antigens, improving treatment precision, and minimizing damage to normal tissues. However, drug resistance and limited efficacy in some patient populations are challenging.

Honokiol, combined with mAbs, has shown the potential to overcome these limitations by enhancing therapeutic responses and mitigating resistance mechanisms. For example, cetuximab is an anti-epidermal growth factor receptor (EGFR) monoclonal antibody approved for treating head and neck squamous cell carcinoma (HNSCC) and metastatic colorectal cancer. Despite its efficacy, resistance to cetuximab frequently develops. Pearson *et al.*^52^ demonstrated that combining honokiol with cetuximab produced significant antiproliferative effects in cetuximab-resistant cancer cells.^53^ The combination downregulated the human epidermal growth factor receptor (HER) family members and inhibited associated signaling pathways, including MAPK and AKT. Furthermore, honokiol reduced the phosphorylation of DRP1 and levels of ROS, indicating altered mitochondrial function. The combination therapy was also validated in cetuximab-resistant HNSCC patient-derived xenograft models, where it led to a notable delay in tumor growth and decreased activation of MAPK, AKT, and DRP1 signaling, consistent with *in vitro* findings.

These results highlight honokiol’s potential to overcome resistance to cetuximab and enhance the efficacy of mAb-based therapies. In addition, honokiol’s ability to modulate key signaling pathways and counteract resistance mechanisms supports its use as a promising adjunct in combination therapies involving mAbs and other targeted agents.

## 4. Honokiol in combination with small-molecule inhibitors (SMIs)

SMIs play a central role in modern oncology, offering targeted inhibition of signaling proteins and pathways critical to tumor growth and survival.^54^ They effectively block RTKs, such as EGFR, mesenchymal-epithelial transition factor (MET), and vascular endothelial growth factor receptors (VEGFR), intracellular signaling mediators, such as MAPK kinase and PI3K, and apoptotic regulators, including B-cell lymphoma 2.^55^ However, challenges such as acquired resistance and toxicity limit their long-term success. Honokiol has emerged as a promising agent in combination with SMIs, enhancing their anti-tumor efficacy while helping to overcome resistance and reduce side effects.^24,56-58^

### 4.1. Cabozantinib (XL-184) and honokiol

Cabozantinib is a multi-kinase inhibitor targeting c-MET, VEGFRs, and other RTKs and has demonstrated significant efficacy in cancers such as renal cell carcinoma (RCC). Despite its clinical success, tumor resistance often develops, limiting its long-term benefit.

Recent studies from our laboratory investigated the synergistic effects of cabozantinib and honokiol in RCC models. The studies focused on the role of the c-MET RTK in cancer progression and resistance. Hyperactivation of c-MET promotes cancer cell survival by activating pathways that help them withstand oxidative stress, contributing to drug resistance.^26,57^

Mechanistic investigations identified proteins such as Rubicon and p62, which regulate autophagy and oxidative stress, along with the transcription factor nuclear factor erythroid 2-related factor 2, as key players in resistance. The combination of cabozantinib and honokiol significantly inhibited RCC cell proliferation *in vitro* and reduced tumor growth *in vivo* in xenograft models. Moreover, this combination therapy decreased the expression of Rubicon, p62, and heme oxygenase-1, reducing tumor vascular density.^57^

These findings highlight honokiol’s potential to enhance cabozantinib’s anti-tumor efficacy and offer a promising strategy to overcome resistance in RCC treatment.

### 4.2. Lapatinib and honokiol

Lapatinib is a dual tyrosine kinase inhibitor targeting EGFR and HER2, primarily used to treat HER2-positive breast cancer. Despite its efficacy, resistance, and toxicity remain concerns in clinical practice.

Honokiol has demonstrated broad anticancer activity in various breast cancer cell lines, including estrogen receptorpositive, estrogen receptor-negative, and drug-resistant lines (e.g., adriamycin- and tamoxifen-resistant cells).^24^ It induces G1-phase cell cycle arrest and caspase-dependent apoptosis in a time- and dose-dependent manner. Notably, HER2 knockdown increases cellular sensitivity to honokiol-induced apoptosis in HER2-overexpressing cells.

The combination of honokiol and lapatinib significantly amplifies anti-tumor effects in HER2-overexpressing breast cancer models. Mechanistically, honokiol downregulates AKT phosphorylation and upregulates *PTEN* expression, resulting in suppression of the PI3K/AKT/mammalian target of rapamycin (mTOR) pathway, an essential driver of cancer cell survival and proliferation.^24^ These findings support the potential of combining honokiol with lapatinib as a novel strategy for HER2-positive breast cancer.

### 4.3. Imatinib and honokiol

Imatinib is a well-established targeted therapy for chronic myeloid leukemia and gastrointestinal stromal tumors. Despite its success, resistance and incomplete responses necessitate a combination approach.

Wang *et al.*^58^ demonstrated that honokiol induces two distinct forms of cell death in leukemia cells: Paraptosis at lower concentrations (characterized by cytoplasmic vacuolization and endoplasmic reticulum swelling) and apoptosis at higher concentrations. These processes may occur sequentially or in parallel, depending on honokiol dosage.

In addition, honokiol disrupts leukemia cell adhesion to the extracellular matrix in a concentration-dependent manner, potentially reducing metastatic potential. Sequential treatment administering honokiol before imatinib exhibited synergistic effects, enhancing imatinib’s therapeutic efficacy in K562 leukemia cells.^59^

These findings suggest that honokiol’s dual-mode induction of cell death, combining apoptotic and non-apoptotic mechanisms, may offer a novel approach for improving imatinib responses in leukemia treatment.

### 4.4. Erlotinib and honokiol

Erlotinib, an EGFR inhibitor, is widely used to treat HNSCC and NSCLC. However, its long-term efficacy is often limited by the development of resistance, necessitating alternative therapeutic strategies. Leeman-Neill *et al.*^59^ investigated honokiol as a potential therapeutic agent for HNSCC, focusing on its ability to target EGFR signaling. Honokiol inhibited tumor cell proliferation (half maximal effective concentration: 3.3 – 7.4 μM), induced apoptosis, and suppressed key EGFR downstream signaling pathways, including MAPK, AKT, and STAT3. In addition, honokiol enhanced the efficacy of erlotinib, leading to significant tumor growth inhibition *in vivo*.^56^

Another study further demonstrated honokiol’s potential in inhibiting lung cancer cell growth, driven by EGFR deregulation. Honokiol at concentrations 2.5 – 7.5 μM suppressed cell proliferation by up to 93% and induced apoptosis in 61% of EGFR-overexpressing bronchial cells. It also downregulated phosphorylated EGFR, AKT, STAT3, and cell cycle-related proteins within 6 – 12 h of treatment. Interestingly, although honokiol exhibited weaker direct EGFR tyrosine kinase binding compared to erlotinib, its overall antiproliferative and pro-apoptotic effects were stronger, suggesting inhibition of additional critical survival pathways. Furthermore, honokiol sensitized erlotinib-resistant cells to erlotinib and significantly reduced lung tumor size and multiplicity by 49% in mouse models. These findings suggest honokiol’s potential as both a monotherapy and an adjuvant strategy for overcoming erlotinib resistance in EGFR-driven cancers.^60^

### 4.5. Osimertinib and honokiol

Osimertinib is a third-generation, Food and Drug Administration-approved EGFR inhibitor that targets *EGFR*-T790M mutations in NSCLC. Despite its clinical success, resistance develops, often due to additional mutations such as C797S, posing a major therapeutic challenge. Honokiol has shown promise in overcoming acquired resistance to osimertinib. In pre-clinical studies, the combination of honokiol and osimertinib synergistically reduced cell viability and colony formation in osimertinib-resistant NSCLC cell lines. This combination also significantly enhanced apoptosis compared to either agent alone.

In mouse xenograft models harboring *EGFR* 19del, T790M, and C797S triple mutations, co-treatment with honokiol and osimertinib effectively suppressed tumor progression. Importantly, the combination was well-tolerated, with no significant toxicity observed in the treated mice. Mechanistic analyses revealed that the combination therapy inhibited phosphorylation of extracellular signal-regulated kinase (ERK) 1/2 and promoted degradation of anti-apoptotic protein myeloid cell leukemia-1, leading to enhanced induction of apoptosis.^61^ These findings support further clinical evaluation of honokiol and its derivatives as adjuvants to overcome osimertinib resistance in *EGFR*-mutant NSCLC.

## 5. Honokiol as an anti-inflammatory agent

Inflammation plays a dual role in disease development, particularly in cancer. Chronic inflammation can be pro-tumorigenic due to the sustained presence of pro-inflammatory cytokines, which promote tumor cell proliferation, survival, angiogenesis, and metastasis. Conversely, acute inflammation can exert anti-tumorigenic effects by enhancing immune surveillance, promoting tumor-associated antigen presentation, and influencing immune cell polarization. Honokiol has been extensively studied for its potent anti-inflammatory properties, contributing to its anti-cancer effects. It inhibits the production of key pro-inflammatory cytokines, including tumor necrosis factor-alpha, IL-1 beta, and IL-6, across various cell types.^62-64^ In addition, honokiol attenuates the activation of critical inflammatory signaling pathways, particularly NF-κB, a key regulator of inflammation. By inhibiting protein kinase C and MAPKs, honokiol disrupts phosphorylation events essential for inflammatory signaling cascades.^65,66^ These properties make honokiol a compelling candidate for modulating tumor-associated inflammation and enhancing the efficacy of anti-cancer therapies.

## 6. Post-transplantation cancer and the role of honokiol in its prevention

Post-transplantation cancers are malignancies that develop in organ or hematopoietic stem cell transplant recipients, primarily due to prolonged immunosuppressive therapy aimed at preventing graft rejection. These therapies, while essential for transplant success, compromise immune surveillance and increase susceptibility to oncogenic viruses and malignancies such as skin cancers, Kaposi sarcoma, and lymphomas, including post-transplant lymphoproliferative disorders^67^ Oncogenic viruses, such as Epstein-Barr virus, human papillomavirus, and human herpesvirus 8 are frequently implicated in these malignancies.^68,69^ Other factors, such as recipient age at the time of transplantation, gender, and genetic pre-disposition, further modulate cancer risk.^70,71^ The present management strategies emphasize regular cancer screening, modulation of immunosuppressive regimens, targeted therapies, and vaccinations against oncogenic viruses.^72,73^

Honokiol has demonstrated potential as an adjuvant therapy to mitigate the increased cancer risk associated with post-transplant immunosuppression. Its anti-inflammatory, antiproliferative, and immunomodulatory properties make it an attractive candidate for integration into post-transplant cancer prevention strategies.

### 6.1. Cyclosporine A and honokiol

Cyclosporine A (CsA) is a calcineurin inhibitor widely used to prevent transplant rejection. It blocks the translocation of the nuclear factor of activated T cells to the nucleus, suppressing T cell activation and immune responses. However, CsA also promotes tumor progression by activating oncogenic pathways such as Ras-Raf-ERK and vascular endothelial growth factor signaling. Our research demonstrated that honokiol, administered alone or in combination with CsA, effectively inhibits these cancer-promoting pathways in RCC models.^74^ Moreover, honokiol’s anti-inflammatory effects may allow for dose reduction of CsA without compromising graft survival, potentially reducing its oncogenic side effects.

### 6.2. Rapamycin and honokiol

Rapamycin (sirolimus), an mTOR inhibitor, is frequently employed to prevent organ rejection, particularly in renal transplantation. While rapamycin possesses inherent anti-tumor activity, prolonged treatment can activate compensatory survival pathways. Specifically, sustained rapamycin exposure relieves the negative feedback loop on AKT through inhibition of S6-kinase, potentially promoting tumor growth through PI3K-mTOR signaling.^75,76^

Sabarwal *et al.*^26^ explored the therapeutic potential of combining honokiol with rapamycin in post-transplantation cancer models. This combination effectively inhibited the c-MET-driven proliferation of renal cancer cells. c-MET is a RTK commonly overexpressed in RCC and linked to tumor growth and metastasis. In addition, the combination downregulated programmed death-ligand 1, a key immune checkpoint molecule that facilitates tumor immune evasion.^26^

In a murine model of post-transplant renal cancer, the honokiol and rapamycin combination prolonged allograft survival and significantly inhibited tumor growth.^77^ Mechanistically, this therapy modulated the expression of tumor-promoting regulators such as Carabin and Rubicon, induced autophagic and apoptotic cell death, and reduced the expression of the RTK AXL, reported to be overexpressed in various cancer types.^78^ Notably, the combination also suppressed the expression of heme oxygenase-1, a cytoprotective enzyme implicated in therapeutic resistance. These findings highlight honokiol’s potential as a novel adjunct therapy to mitigate post-transplant cancer risk while preserving graft survival.

## 7. Conclusion

Therapeutic resistance remains one of the most significant obstacles to effective cancer treatment, contributing to disease progression and treatment failure. In response, numerous therapeutic strategies have been developed to overcome this challenge. These include novel targeted therapies such as SMIs of RTKs, immune checkpoint inhibitors, and mAbs designed to specifically target resistant cancer subtypes. While these agents often elicit promising initial responses, they frequently lead to the emergence of more aggressive and therapy-resistant tumor clones.^79^ Acquired resistance is primarily driven by the complex and adaptive nature of tumor architecture. Tumor cells dynamically remodel their microenvironment through physical and biochemical mechanisms, promoting immune evasion, migration, invasion, and resistance to apoptosis.^80^ These adaptive changes create barriers to effective drug delivery and foster the survival of drug-resistant cancer cell populations. Combination therapies (Table 1) have emerged as a more effective strategy than single-agent treatments, as they simultaneously target multiple oncogenic pathways and enhance tumor cell eradication. Several combination regimens have already gained approval and are in clinical use, although further improvements in efficacy, safety, and tolerability are still needed.^81-83^

In this context, drug repurposing has gained traction as a viable strategy to reduce drug development costs and accelerate the translation of therapies into clinical practice. Natural compounds, including plant-derived bioactive molecules, have been extensively studied for their anticancer potential. Honokiol, in particular, has demonstrated potent anticancer activity across various malignancies, with additional preventive benefits.^84^ Notably, honokiol has shown the ability to sensitize therapy-resistant cancer cells when used in combination with other conventional and targeted treatments.^29,36,38,42,49,53,59,60^ Pre-clinical studies from our laboratory have further confirmed the therapeutic efficacy of honokiol in both cancer and post-transplantation settings. However, to fully elucidate its clinical potential, more in-depth investigations are warranted, including comprehensive pre-clinical studies to fully evaluate the potential of honokiol as a treatment option.

## Figures and Tables

**Table 1.** Combination treatments with honokiol

Drug/therapeutic name	Cancer type/models	Key findings	References
Chemotherapeutic drugs			
Cisplatin	Colorectal cancer, ovarian cancer, oral cancer, lung cancer, and renal cell carcinoma	Reduce toxicity, re-sensitization, interleukin-6/STAT3 regulation, dynamin-related protein 1 regulation, and reactive oxygen species and anti-oxidative enzyme regulation	19,28-34
Paclitaxel (Taxol)	Human squamous KB cells, lung cancer, and breast cancer	Inhibit cell proliferation and tumor growth, induce paraptosis, downregulation of focal adhesion kinase, PI3K, MMP-2, and MMP-9	19,37-40
Doxorubicin	Breast cancer and cardiomyopathy	Inhibit growth and proliferation by regulating microRNA-188-5p, *FBXW7*, and c-*Myc*, regulation of mucin 1 and multidrug resistance protein 1, and cardioprotective properties	42-45
5-fluorouracil	Oral cancer, urothelial cell carcinoma, and tongue cancer	High apoptosis, suppresses tumor growth, cell cycle arrest, and decreased systemic toxicity	47-49
Metformin	Breast cancer	Induce apoptosis and inhibit cell growth	50
Bleomycin	Breast cancer, pancreatic cancer, and melanoma	Reduce pulmonary toxicity and inhibit DNA repair	52
Monoclonal antibodies			
Cetuximab	Cetuximab-resistant cancer cells	Resensitization, regulate HER, MAPK, AKT, and dynamin-related protein 1 pathways	53
Small-molecule inhibitors			
Cabozantinib	Renal cell carcinoma	Induce reactive oxygen species-mediated apoptosis and autophagy, inhibit Rubicon, p62, and HO-1	57
Lapatinib	Breast cancer	Cell cycle arrest induces apoptosis, suppresses PI3K/AKT/mTOR pathway	24
Imatinib	Leukemia	Inhibit cell adhesion to the extracellular matrix and induce paraptosis	59
Erlotinib	Head and neck squamous cell carcinoma and lung cancer	Induce apoptosis, inhibit EGFR signaling pathways, including MAPK, AKT, and STAT3	56,60
Osimertinib	Non-small cell lung cancer	Inhibit cell proliferation and induce apoptosis, suppress tumor growth even with 19del, T790M, and C797S triple mutations, inhibit p-ERK1/2, and promote myeloid cell leukemia-1 degradation	61
Immunosuppressive drugs in transplantation			
Cyclosporine A	Renal cell carcinoma	Inhibit cyclosporine A-induced Ras-Raf-ERK and VEGF pathways	74
Rapamycin	Renal cell carcinoma	Inhibit cell proliferation and growth, inhibit Rubicon, programmed death-ligand 1, c-mesenchymal-epithelial transition factor, and AXL, and downregulate HO-1	26,77

Abbreviations: AKT: Protein kinase B; EGFR: Epidermal growth factor receptor; ERK: Extracellular signal-regulated kinase; HER: Human epidermal growth factor receptor; HO-1: Heme oxygenase-1; MAPK: Mitogen-activated protein kinase; MMP: Matrix metallopeptidase; mTOR: Mammalian target of rapamycin; PI3K: Phosphoinositide 3-kinase; STAT3: Signal transducer and activator of transcription 3; VEGF: Vascular endothelial growth factor.
